# Clinical application of intubation-free anesthesia in radical resection of lung cancer

**DOI:** 10.3389/fmed.2023.1175437

**Published:** 2023-05-15

**Authors:** Zhenhai Liu, Shengjie Ren, Ning Liu, Yanhua Luo

**Affiliations:** ^1^Department of Anesthesiology, Weifang People’s Hospital, Weifang, China; ^2^School of Anesthesiology, Weifang Medical University, Weifang, China

**Keywords:** intubation-free anesthesia, VATS, adverse reactions, radical resection of lung cancer, airway management protocol

## Abstract

**Background:**

In recent years, video-assisted thoracic surgery (VATS) has become increasingly widespread. However, the implementation of VATS requires the assistance with lung isolation techniques. Spontaneous breathing with laryngeal masks is also increasingly used in VATS. However, evidence on the characteristics of intubated anesthesia is insufficient.

**Objective:**

This study aimed to explore whether intubation-free anesthesia has more advantages than other intubation methods in the clinical setting.

**Methods:**

Patients with lung tumors who underwent VATS in our hospital between June 2022 and October 2022 were included in the study. Perioperative data of patients, including basic information, intraoperative hemodynamic changes, postoperative inflammatory indicators, and adverse reactions were obtained through the electronic medical record system. According to the protocol of airway management during anesthesia, participants were divided into the following groups: laryngeal mask with spontaneous breathing group (LMSB group), laryngeal mask combined with bronchial blocker group (LM + BB group), double-lumen tube group (DLT group), and tracheal tube combined with bronchial blocker group (TT + BB group). All data were analyzed using SPSS 25.0 software.

**Results:**

At baseline, patients in the LMSB and LM + BB groups had a lower body weight (*P* = 0.024). Systolic blood pressure (SBP), diastolic BP (DBP), and heart rate (HR) were significantly higher in the DLT group than in the non-intubated group during surgery (SBP: T1 *P* = 0.048, T4 *P* = 0.021, T5 *P* ≤ 0.001, T6 *P* ≤ 0.001, T7 *P* = 0.004; DBP: T5 *P* ≤ 0.001, T6 *P* ≤ 0.001, T7 *P* ≤ 0.001; HR: T1 *P* = 0.021, T6 *P* ≤ 0.001, T7 *P* = 0.007, T8 *P* ≤ 0.001). The input fluid (*P* = 0.009), urine output (*P* = 0.010), surgery duration (*P* = 0.035), and procalcitonin levels (*P* = 0.024) of the DLT group were also significantly higher than those of the other groups. The recovery duration of the LMSB group was significantly longer (*P* = 0.003) and the incidence of postoperative adverse reactions, mainly atelectasis, was higher (*P* = 0.012) than those of the other groups.

**Conclusion:**

Although the intubation-free anesthesia has less stimulation during operation and less postoperative inflammatory response, it has obvious adverse reactions after operation, which may be not the best anesthesia scheme for radical resection of lung cancer in VATS.

**Clinical trial registration:**

https://www.chictr.org.cn/showproj.html?proj=182767, identifier ChiCTR2200066180.

## Introduction

With the prevalent use of computed tomography for screening and the increase in the aging population, the incidence of lung cancer is increasing annually, especially in women (1, 2). For small cell lung cancer, the oncologic outcome varies greatly, ranging from a few months to several years (3). Fortunately, because of earlier detection (4) and improved medical care (5), the lung cancer-associated mortality is decreasing. The advent of video-assisted thoracic surgery (VATS) has saved patients with lung cancer from the pain of thoracotomy (6). A great diversity of lung isolation techniques is required in VATS to protect the healthy lung from the affected lung and provide a clear surgical field. A bronchial blocker appears to be less damaging to the airway than a double lumen; however, its placement is difficult (7, 8). Each lung isolation technique has its own advantages and disadvantages, and which lung isolation technique is more advantageous remains controversial.

Laryngeal masks for spontaneous breathing is also increasingly applied in VATS (9). Spontaneous breathing during anesthesia may reduce the injury incurred from mechanical ventilation; however, it cannot achieve sharp a surgical field, which may not be satisfactory for the surgeon. The use of a laryngeal masks for spontaneous breathing in VATS requires shallow breathing; thus, hypercapnia, or even hypoxemia, is likely to occur. There is no consensus on whether intubation-free anesthesia, like laryngeal masks for spontaneous breathing, is better than pulmonary isolation under thoracoscopic lobectomy. This study aimed to explore whether intubation-free anesthesia has more advantages than other intubation methods and whether it is suitable for further promotion.

## Materials and methods

### Setting and patients

This study has been approved by the Ethics Committee of Weifang People’s Hospital (KYLL20221125-1) and has been registered with the China Clinical Experimental Center (ChiCTR2200066180). Patients with lung tumors who underwent thoracoscopic lobectomy in our hospital between June and October 2022 were retrospectively included. Inclusion criteria included patients aged 18–60 years, with American Society of Anesthesiologists (ASA) grades I–III. Patients who had contraindications for the lung isolation technique, such as aneurysms, anterior mediastinal masses, idiophathic bronchial stenosis and bronchial anatomy variations etc., those with severe cardiopulmonary insufficiency and liver and kidney dysfunction, and those planning to undergo a second operation within 7 days of surgery or transfer treatment were excluded from this study. According to the methods of airway management during anesthesia, participants were divided into laryngeal mask with spontaneous breathing (LMSB group), laryngeal mask combined with bronchial blocker (LM + BB group), double-lumen tube (DLT group), and tracheal tube combined with bronchial blocker (TT + BB group) groups.

### Anesthesia and postoperative treatment

All the anesthesia and surgery in this study was performed by the same anesthesiologist and surgeons. After entering the operating room, an intravenous line was inserted for all included patients, and their electrocardiogram, non-invasive blood pressure, pulse oximetry, P_*ET*_CO_2_, and bispectral index (BIS) levels were monitored. Invasive arterial blood pressure was obtained through a radial artery puncture. The patient underwent lung isolation technique during anesthesia, and the following anesthesia protocol was used. Preoxygenation was administered for 3 min before anesthesia. Anesthesia induction was achieved with propofol (plasma concentration: 5 μg/mL), remifentanil (1.0 μg/kg), sufentanil (20 μg), and rocuronium (0.6 mg/kg). When the medication effects set in, a DLT or TT + BB was inserted, and fiberoptic bronchoscope was used to confirm the position. After the patient changed to the lateral decubitus position, 0.375% bupivacaine 20 ml was used for ultrasound-guided serratus anterior block. To compensate for the incomplete serratus anterior block, intercostal nerve block was performed under thoracoscopy with 0.375% bupivacaine 2 ml per plane. Anesthesia was maintained with remifentanil (0.1 μg/kg/min) and propofol (plasma concentration: 2.5 μg/mL). Pure oxygen inhalation was maintained prior to one-lung ventilation (OLV), which was initiated after cutting the pleura, and the tidal volume (6–8 mL/kg) and respiration rate (12–16 beats per min) were adjusted accordingly. The operation was completed after no problems with mechanical inflation of the lung occurred. The DLT or TT + BB was withdrawn when the patient reached the indication. The patient entered the postanesthesia care unit with an analgesic pump (hydromorphone 8 mg + normal saline 100 mL, pumped at a rate of 2 mL/h).

The anesthesia protocol for patients breathing spontaneously with a laryngeal mask was as follows. Patients were administered with 50 μg of dexmedetomidine as a premedication. Propofol (plasma concentration: 4 μg/mL) and sufentanil (0.5–1 μg/kg) were used for inducing anesthesia. A size 4 laryngeal mask was inserted after sufficient induction of anesthesia. Then, the dose of the drug was reduced for the resumption of spontaneous breathing. Based on the serratus anterior and intercostal nerve block, local pulmonary surface anesthesia with 2% lidocaine 10 ml and vagus nerve block with 2% lidocaine 2–5 ml were performed under direct vision to reduce cough. Anesthesia was maintained with propofol (plasma concentration: 2 μg/mL) and remifentanil (0.03–0.05 μg/kg/min), and BIS was maintained at 40–60. The analgesic pump and postoperative treatment were the same as those in the lung isolation group. The medication plan of the LM + BB group was the same as that of the DLT group. The sequence of intubation was to insert the laryngeal mask to the appropriate position first and to insert the BB with the assistance of a fiberoptic bronchoscope subsequently.

### Data collection

The following data were collected and recorded through the electronic medical record system. (i) Demographics: sex, age, weight, ASA classification and Charlson Score (a comorbidity index). (ii) Intraoperative hemodynamic data: The time points before and after induction of anesthesia were denoted as T1 and T2, and 5 min, 10 min, 30 min, 1 h, 2 h, and 3 h after induction of anesthesia were expressed as T3–T8. The systolic blood pressure (SBP), diastolic blood pressure (DBP), and heart rate (HR) were recorded at these eight time points. (iii) Postoperative information: Inflammatory indicators (hemameba, neutrophils, C-reactive protein, and procalcitonin) and adverse reactions (hypoxemia, arrhythmia, massive hemorrhage, pulmonary infection, and atelectasis). Hypoxemia was defined as a blood oxygen saturation of less than 90%. Arrhythmia refers to the abnormal electrical conduction system of the heart caused by irregular heartbeat, too fast or too slow symptoms. Massive blood loss was defined as blood loss greater than 200 ml per day. Pneumonia is diagnosed with a suspected respiratory infection treated with antibiotics and at least one of the following: new or change of pulmonary opacities, white blood cell count > 12000/mm^3^, body temperature > 38.5°C, and positive sputum culture. The presence of atelectasis in the chest x-ray was considered as atelectasis. (iv) Surgery information: Volume of fluid infusion, blood loss, and urine output; duration of recovery and surgery; drainage tube and postoperative hospital stay; and anesthesia and total hospital cost.

### Statistical analyses

All data in this study were analyzed using SPSS25.0 (IBM Corp). Continuous data were analyzed according to the normality test of p-p diagram. We determined whether the data conformed to normal distribution. The normally distributed data are indicated as means ± standard deviations, whereas non-normally distributed data are expressed as medians (interquartile ranges). For data that satisfied the homogeneity of variance and conformed to the normal distribution, one-way analysis of variance was used to compare the differences among the four groups; otherwise, Kruskal–Wallis test was used. All categorical data are represented as frequencies (proportions) and were analyzed using the χ2 test or Fisher’s precision probability test, as appropriate. A *P*-value < 0.05 was considered statistically significant.

## Results

**FIGURE 1:**
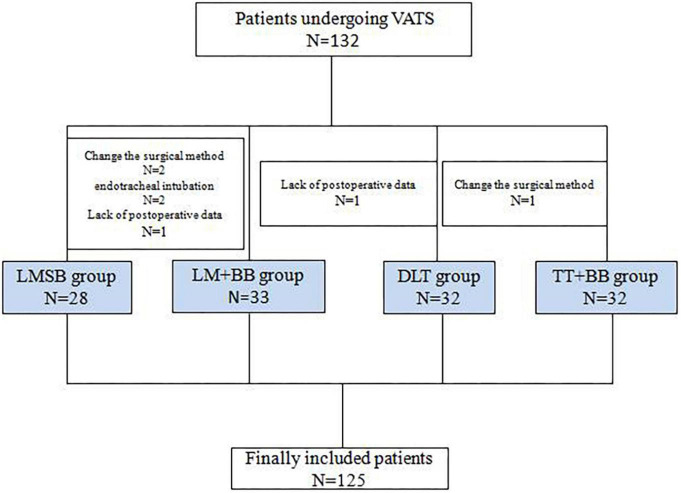
Study flow. VATS, video-assisted thoracic surgery.

A total of 132 patients underwent thoracoscopic lobectomy in our hospital between June 2022 and October 2022, and 125 patients were finally included in the study. As shown in Figure 1, patients dropped out of the study because the surgical method (*N* = 3) and endotracheal intubation (*N* = 2) were changed, and for two patients, postoperative data were lost. Overall, the four groups were well-matched for the baseline characteristics as shown in Table 1, except for obvious statistical differences in weight (*P* = 0.024, LMSB group vs DLT group *P* = 0.039, LMSB group vs TT + BB group *P* = 0.012, LM + BB group vs TT + BB group *P* = 0.026).

**TABLE 1:** Characteristics of patients at baseline.

	LMSB group	LM + BB group	DLT group	TT + BB group	*P*-value
Female, *n* (%)	13 (46.43)	19 (57.58)	17 (53.13)	21 (65.62)	0.496
Male, *n* (%)	15 (53.57)	14 (42.42)	15 (46.87)	11 (34.38)	
Age, year	51.68 ± 12.96	54.84 ± 14.16	54.37 ± 7.32	56.09 ± 7.64	0.468
Weight, kg	62.69 ± 9.36^ab^	63.73 ± 10.23^c^	68.33 ± 12.16^a^	69.38 ± 8.41^bc^	0.024
**ASA classification**					
I grade, *n* (%)	21 (75.00)	23 (69.69)	23 (71.87)	17 (53.12)	0.091
II grade, *n* (%)	6 (21.43)	10 (30.31)	7 (21.88)	9 (28.13)	
III grade, *n* (%)	1 (3.57)	0 (0.00)	2 (6.25)	6 (18.75)	
Charlson score	0.21 ± 0.09	0.30 ± 0.08	0.25 ± 0.07	0.44 ± 0.10	0.308
Hemameba, *10^9^/L	6.09 ± 1.61	6.46 ± 1.88	6.01 ± 1.40	6.45 ± 1.61	0.612
Neutrophils, *10^9^/L	3.81 ± 1.35	3.95 ± 1.80	3.66 ± 1.39	3.72 ± 1.09	0.895
CRP, mg/L (range)	0.60 (0.38, 0.83)	0.80 (0.50, 1.75)	0.50 (0.50, 1.37)	0.50 (0.50, 2.40)	0.560
Procalcitonin, ng/mL	0.01 (0.01, 0.02)	0.01 (0.01, 0.02)	0.02 (0.01, 0.05)	0.02 (0.01, 0.04)	0.089

Values are means ± SDs, numbers (%), or medians and ranges. ASA, American Society of Anesthesiologists; CRP, C-reactive protein.

^a^LMSB group vs DLT group *P* = 0.039.

^b^LMSB group vs TT + BB group *P* = 0.012.

^c^LM + BB group vs TT + BB group *P* = 0.026.

**FIGURE 2:**
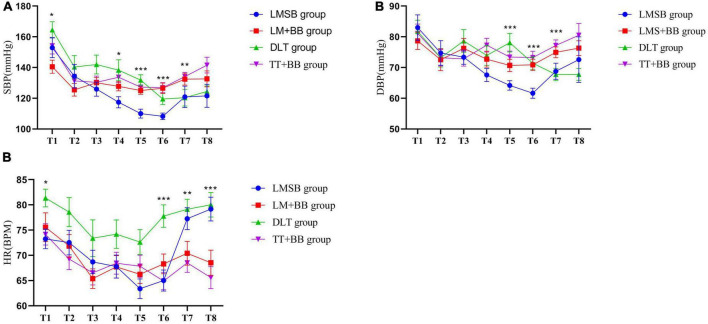
Intraoperative hemodynamic changes. **(A)** SBP T1:*P = 0.048; T4:*P = 0.021; T5:***P ≤ 0.001; T6:***P ≤ 0.001; T7:**P = 0.004. **(B)** DBP T5:***P ≤ 0.001; T6:***P ≤ 0.001; T7:***P ≤ 0.001. **(C)** HR T1:*P = 0.012; T6:***P ≤ 0.001; T7:**P = 0.007; T8:***P ≤ 0.001.

The trend changes of hemodynamics in the four groups at eight time points are shown in Figure 2. The SBP (Figure 2A) of the four groups showed statistical differences 10 min after anesthesia induction (T1 *P* = 0.048, T4 *P* = 0.021, T5 *P* ≤ 0.001, T6 *P* ≤ 0.001, T7 *P* = 0.004), whereas the discrepancy of DBP (Figure 2B) and HR (Figure 2C) started 30 min after anesthesia induction (DBP: T5 *P* ≤ 0.001, T6 *P* ≤ 0.001, T7 *P* ≤ 0.001; HR: T1 *P* = 0.021, T6 *P* ≤ 0.001, T7 *P* = 0.007, T8 *P* ≤ 0.001). It was clear that the blood pressure level of the LMSB group was significantly lower than those of the other three groups, and the HR of the DLT group was higher than those of the other three groups during surgery.

In terms of intraoperative volume (Table 2), the volume of the input fluid in the DLT group was significantly higher than that in the LMSB group and LM + BB group (*P* = 0.009, LMSB group vs DLT group *P* ≤ 0.001, LM + BB group vs DLT group *P* = 0.040), and a similar trend was observed in urine output (*P* = 0.010, LMSB group vs DLT group *P* = 0.009, LM + BB group vs DLT group *P* = 0.019). However, there was no significant difference in the intraoperative blood loss.

**TABLE 2:** Intake and output volume.

	LMSB group	LM + BB group	DLT group	TT + BB group	*P*-value
Input fluid (ml)	1071.42 @ 352.61^a^	1196.96 @ 248.10^b^	1375.00 @ 359.21ab	1206.25 @ 409.51	0.009
Blood loss (ml)	53.69 @ 121.67	61.03 @ 70.93	65.46 @ 89.473	63.10 @ 73.02	0.969
Urine volume (ml)	246.67 @ 178.13^c^	264.81 @ 111.64^d^	355.17 @ 161.10^cd^	296.29 @ 112.59	0.010
Chest drainage volume (ml)	497.69 @ 482.11	564.48 @ 545.30	538.06 @ 307.71	656.03 @ 801.94	0.378

Values are means ± SDs.

^a^LMSB group vs DLT group *P* ≤ 0.001.

^b^LM + BB group vs DLT group *P* = 0.040.

^c^LMSB group vs DLT group *P* = 0.009.

^d^LM + BB group vs DLT group *P* = 0.019.

**FIGURE 3:**
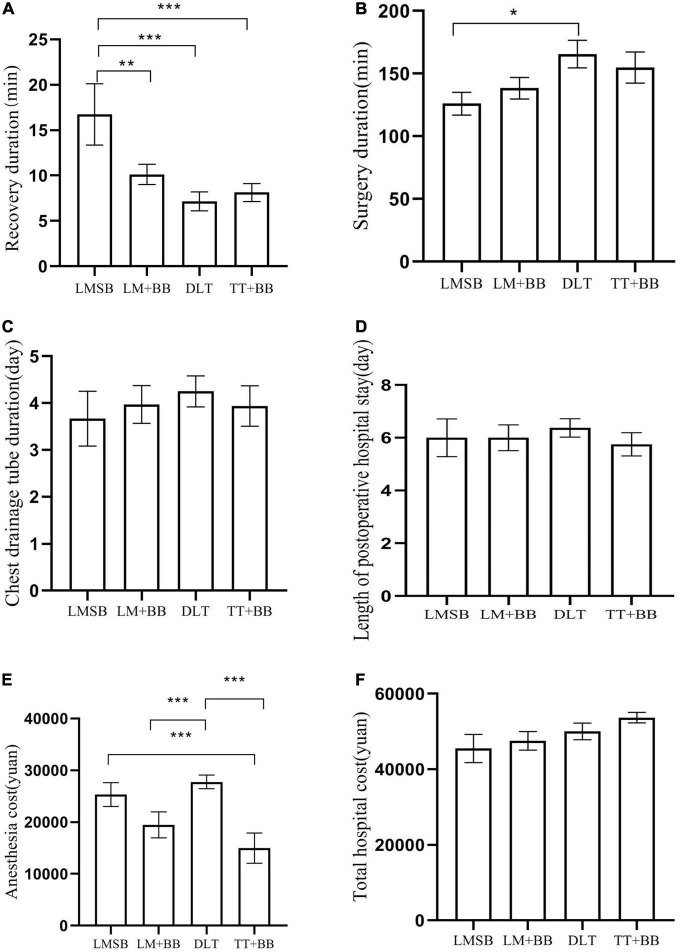
Surgical information. **(A)** Recovery duration. It is the time from drug withdrawal to patient’s recovery of consciousness. ***LMSB group vs DLT group *P* ≤ 0.001, ***LMSB group vs TT + BB group *P* ≤ 0.001, **LMSB group vs LM + BB group *P* = 0.007. **(B)** Surgery duration, that is, the time from the beginning of skin cutting to the end of skin suturing. *LMSB group vs DLT group *P* = 0.011. **(C)** Chest drainage tube duration. The chest drainage tube is removed when the patient’s lungs are well distended and there is no gas or fluid out flow. **(D)** Length of postoperative hospital stay. Length of hospital stay before surgery is not included. **(E)** Anesthesia cost. ***LMSB group vs TT + BB group *P* = 0.003, LM + BB group vs DLT group *P* = 0.006, and DLT group vs TT + BB group *P* ≤ 0.001. **(F)** Total hospital cost.

It can be observed from Figure 3A that the recovery duration of the LMSB group was the longest among the four groups (*P* = 0.003, LMSB group vs LM + BB group *P* = 0.007, LMSB group vs DLT group *P* ≤ 0.001, LMSB group vs TT + BB group *P* ≤ 0.001), whereas the surgery duration of LMSB group was the shortest (*P* = 0.035, LMSB group vs DLT group *P* = 0.011) (Figure 3B). No significant statistical differences were found between the four sets of data in the aspects of drainage tube duration (Figure 3C) and length of postoperative hospital stay (Figure 3D). Although there was no difference in total hospital costs (Figure 3F), the statistical difference in anesthesia costs among the four groups is obvious (LMSB group vs TT + BB group *P* = 0.003, LM + BB group vs DLT group *P* = 0.006, and DLT group vs TT + BB group *P* ≤ 0.001) (Figure 3E).

Among the inflammatory response indicators at 48 h after surgery (Table 3), only procalcitonin showed significant differences between groups (*P* = 0.024, LMSB group vs DLT group *P* ≤ 0.001, LMSB group vs TT + BB group *P* = 0.003). In other inflammatory indicators, the DLT group was also found to have higher levels than the other groups, although there was no statistical difference. However, it is indicated in Table 4 that the incidence of postoperative adverse reactions was higher in the LMSB group and LM + BB group, especially atelectasis (*P* = 0.012).

**TABLE 3:** Inflammatory indicators at 48 h after surgery.

	LMSB group	LM + BB group	DLT group	TT + BB group	*P*-value
Hemameba, *10^9^/L	9.06 ± 2.51	8.81 ± 2.63	9.67 ± 2.86	9.11 ± 3.05	0.710
Neutrophils, *10^9^/L	6.94 ± 2.36	6.36 ± 1.96	7.49 ± 2.69	6.84 ± 2.58	0.459
CRP, mg/L (range)	37.60 (20.80, 51.60)	46.10 (31.40, 69.20)	50.30 (26.82, 70.40)	43.70 (14.25, 83.40)	0.560
Procalcitonin, ng/mL	0.02^ab^ (0.01, 0.32)	0.06 (0.01, 0.19)	0.14^a^ (0.11, 0.26)	0.08^b^ (0.04, 0.16)	0.001

Values are means ± SDs, and medians and ranges. CRP, C-reactive protein.

^a^LMSB group vs. DLT group *P* ≤ 0.001.

^b^LMSB group vs. TT + BB group *P* = 0.003.

**TABLE 4:** Postoperative complications.

	LMSB group	LM + BB group	DLT group	TT + BB group	*P*-value
Hypoxemia, *n* (%)	0 (0.00)	2 (6.06)	0 (0.00)	0 (0.00)	0.245
Arrhythmology, *n* (%)	1 (3.57)	2 (6.06)	0 (0.00)	1 (3.12)	0.738
Massive blood loss, *n* (%)	1 (3.57)	2 (6.06)	0 (0.00)	0 (0.00)	0.308
Pulmonary infection, *n* (%)	8 (28.57)	12 (36.36)	13 (40.62)	8 (25.00)	0.534
Pulmonary atelectasis					
Mild level, *n* (%)	5 (17.86)	5 (15.15)	1 (3.12)	0 (0.00)	0.012
Moderate level, *n* (%)	1 (3.57)	0 (0.00)	0 (0.00)	0 (0.00)	

Values are numbers (%).

## Discussion

Our study revealed that the intubation-free anesthesia has less stimulation during operation and less postoperative inflammatory response, but it has obvious adverse reactions after operation, such as atelectasis, which may be not the best anesthesia scheme for radical resection of lung cancer underwent thoracoscopic lobectomy. The stimulation of DLT is strong, which may cause postoperative inflammatory response. Moreover, the TT + BB, one of the airway management methods, may be superior to other approaches.

The general consensus is that endotracheal intubation is often an essential part of the rescue process; however, it also causes stress and other adverse events (10), which may further lead to endocrine and metabolic changes (11). The stress induced by an insufficient homeostatic capacity will cause inflammation (12). Moreover, the inflammatory responses under acute stress are indeed valuable in predicting the development of long-term changes (13). The stress response caused by the DLT contributes to hemodynamic perturbations and postoperative hoarseness of voice and sore throat (14, 15). When the position was changed from supine to lateral decubitus during thoracic surgery, proper bronchial cuff pressure of DLT will rise and cause further stress stimulation (16). It was also found in our study that the DLT caused a large intraoperative blood pressure fluctuation, rapid heart rate, and relatively obvious postoperative inflammatory response, whereas intubation-free anesthesia is quite the opposite. Thus, more liquid capacity is needed to keep the internal environment stable.

General anesthesia with OLV used to be generally considered mandatory for VATS procedures. Recently, to reduce the risk of endotracheal intubation (10), laryngeal mask was employed in VATS (9, 17, 18). Consistent with our research, non-intubated methods of anesthesia may be more appropriate for patients with low body weight (19). The lower weight of patients in the non-intubation group may be the reason for the difference in SBP and HR before operation. VATS without tracheal intubation has many advantages, such as a short hospital length of stay, faster recovery, and less short-term complications (20, 21). However, due to the relative instability of the laryngeal mask, patients who are expected to have a long operation time will also avoid using it, which may be one of the limitations of this study.

However, there are several problems that must be addressed with VATS under non-intubated anesthesia (22). Firstly, laryngeal mask ventilation, especially the LMSB, must require high-frequency and small tidal volume to clear the surgical field, which may easily generate hypercapnia (23). The five patients in the LMSB group experienced delayed recovery, and the recovery duration of the LMSB group is the longest. Hypercapnia was suspected as one of the reasons, and the sample size may have influenced the results. Secondly, the patients with laryngeal mask (including LMSB and LM + BB) had a higher incidence of postoperative atelectasis. This was attributed to the inability to fully inflate the lungs at the end of surgery (22). Achieving OLV and a clear surgical field are among the advantages of LM + BB over LMSB; however, it renders no access to the non-dependent side of the lung (24, 25). Furthermore, a risk for postoperative incomplete lung inflation still exists. Finally, the laryngeal mask in VATS is more demanding and requires the cooperation of experienced anesthesiologists and surgeons. Therefore, there is no significant advantage to the non-intubation approach with VATS.

It has to be admitted that intubation-free anesthesia is the result of advances in anesthesia for thoracoscopic surgery. The different surgical plans naturally have decisive significance for the prognosis of tumors, but there are still many scholars who have studied intubation-free anesthesia. This non-intubated approach may not be suitable for oncological patients undergoing anatomic sections, but it have obvious advantages for some thinner patients and restrictive procedures, such as surgical lung biopsy, mediastinal biopsy, limited wedge resection (26). Cherchi et al. (27) showed the safety and efficacy of surgical lung biopsy with a intubation-free approach in patients affected by lung interstitiopathy. Maintenance of spontaneous breathing through LMA during VATS lung biopsy is associated with better postoperative diaphragmatic residual function (28) and faster return to normal respiratory physiology (21). For the VATS lung metastasectomy, non-intubated anesthesia produced less immune and inflammatory responses compared to traditional anesthesia (29). These are worthy of our clinical attention and promotion. Although we did not find very meaningful data about non-intubated approach in our research results, it does not hinder us from further research and does not deny the possibility of obtaining new valuable conclusions in further research. Personalized use of intubation methods can give full play to the advantages of various anesthesia programs.

The TT + BB is a traditional OLV mode, which can achieve complete removal of larger tumors and have fewer complications and shorter hospital stays (30). TT + BB overcomes the biggest shortcoming of a double-lumen tube, that is, less stimulation from intubation, and significantly reduces the incidence of postoperative hoarseness and sore throat (31). In the meantime, TT + BB can achieve an ideal lung deflation and inflation effect that reduce the incidence of postoperative adverse events (32). Moreover, it also more cost-efficient. This study recommends that TT + BB is the most optimal airway management modality for VATS, except in special cases.

Our study also has some limitations. Firstly, if the sample size increases, it may be that the study’s conclusions are more authoritative. Secondly, no correlation analysis was performed regarding intraoperative respiratory parameters, such as, airway pressure, end-tidal carbon dioxide pressure, etc. Finally, long-term follow-up was not performed to obtain survival data. These limitations do not affect the validity of our conclusions.

## Conclusion

The intubation-free anesthesia may be not the best anesthesia scheme for radical resection of lung cancer in VATS, which has less stimulation during operation and less postoperative inflammatory response, but it has obvious adverse reactions after operation. The TT + BB is recommended as the optimal airway management protocol for VATS. It overcomes the disadvantages of DLT, with less stimulation from intubation before surgery, stable hemodynamics during surgery, and fewer adverse reactions after surgery. More prospective studies with large samples are required to reinforce this conclusion.

## Data availability statement

The original contributions presented in this study are included in the article/supplementary material, further inquiries can be directed to the corresponding author.

## Ethics statement

The studies involving human participants were reviewed and approved by the Weifang People’s Hospital Ethics Committee. Written informed consent for participation was not required for this study in accordance with the national legislation and the institutional requirements.

## Author contributions

YL designed the study protocol. ZL analyzed the data and wrote the manuscript. SR and NL collected the data. All authors contributed to the article and approved the submitted version.
